# The Emerging Science of BMAA: Do Cyanobacteria Contribute to Neurodegenerative Disease?

**DOI:** 10.1289/ehp.120-a110

**Published:** 2012-03-01

**Authors:** Wendee Holtcamp

**Affiliations:** Houston-based freelancer **Wendee Holtcamp** has written for *Nature*, *Scientific American*, *National Wildlife*, and other magazines.


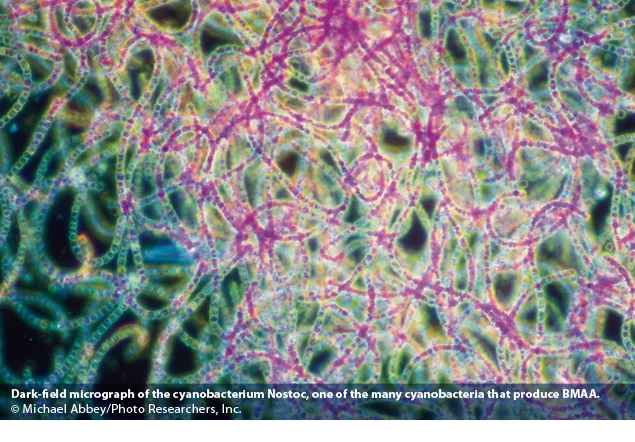
In the late 1990s ethnobotanist Paul Alan Cox visited the indigenous Chamorro people of Guam, sleuthing for cancer cures in the lush rainforest. He soon stumbled upon troubling facts that would change the trajectory of his career, leading to major clues in understanding Lou Gehrig’s disease (amyotrophic lateral sclerosis, or ALS) and possibly other neurodegenerative diseases. Since that time, major breakthroughs in the fields of neurobiology, epidemiology, and ecology have led to an increased interest in an unlikely hypothesis: that β-methylamino-ʟ-alanine (BMAA)—a cyanobacterial neurotoxin found in contaminated seafood and shellfish, drinking water supplies, and recreational waters—may be a major factor in these diseases.

Last fall, writer Wendee Holtcamp visited Paul Cox’s Institute for EthnoMedicine and Yellowstone National Park’s Grand Prismatic Spring, named for the vividly colored cyanobacteria that live along the spring’s edge. She also kayaked on Lake Houston to collect water and sediment, which Cox’s group tested for BMAA. The results of that test were described in the January/February 2012 issue of Miller-McCune Magazine.^55^

## A Trail of Clues

The trail of clues began soon after U.S. forces recaptured Guam from the Japanese in 1944. A Navy neurologist noticed the Chamorro people succumbed to a strange neurodegenerative illness that caused paralysis, shaking, and dementia at 50–100 times the incidence of ALS worldwide.^1^^,^^2^ The illness was dubbed amyotrophic lateral sclerosis–parkinsonism/dementia complex (ALS-PDC), known locally as lytico-bodig. Since then, neurologists have converged on the island to crack the medical version of the world’s hardest math problem. Solving the mystery of this many-faceted illness, it was hoped, could unlock a deeper understanding of neurodegenerative diseases worldwide and possibly lead to a cure.

BMAA was first isolated from cycad trees on Guam in 1967. The discovery was serendipitous, an offshoot of research on lathyrism, a progressive paralysis of the legs found in people in China, India, and the Middle East. Studies had linked lathyrism to consumption of certain species of legumes that contained the compound β-*N*-oxalylamino-ʟ-alanine (BOAA).^3^ Marjorie Whiting, a nutritional anthropologist working on Guam for the National Institutes of Health, recognized a similarity between lathyrism and ALS-PDC and asked Arthur Bell, a noted plant biochemist and director of the Kew Royal Botanic Gardens, to test cycad seeds for BOAA. Although the cycads didn’t contain BOAA, Bell discovered a similar compound with a methyl group instead of an oxalyl group—BMAA.^4^^,^^5^

Subsequent research showed that BMAA caused convulsions in chicks^6^ and rats^7^ and damaged rat neurons.^8^ However, dietary exposure did not cause delayed symptoms in rats,^7^ whereas ALS-PDC, it soon became clear, developed years or even decades after exposure ceased. In the 1980s Peter Spencer, then a neurotoxicologist at the Albert Einstein College of Medicine, briefly resurrected the BMAA hypothesis and reported shaking and paralysis in macaques fed BMAA,^9^ but his work was heavily criticized by another team of neurologists that argued that people would have to eat kilograms of cycad flour to ingest a comparable dose.^10^

Cox arrived in Guam in the late 1990s after the trail had run cold, but through a series of discoveries, he resurrected the dormant hypothesis that BMAA was the cause behind ALS-PDC. The Chamorro made tortillas out of ground cycad seeds, which they washed repeatedly to remove toxins (if their chickens didn’t die after drinking the wash water, the people deemed the seeds safe to grind and eat). They also ate feral pigs and fruit bats that fed on cycad seeds—the bats, known as Mariana flying foxes, were stewed in coconut cream and eaten whole—brains, bones, skin, and all. In 2002 Cox and Columbia University Medical Center neurologist Oliver Sacks hypothesized that chronic dietary exposure creates a neurotoxic reservoir of BMAA in the brain tissues of the Chamorros that, after a lag time, leads to a neuronal meltdown.^11^ The breakthroughs that would expand the story beyond Guam were soon to come.

**Figure:**
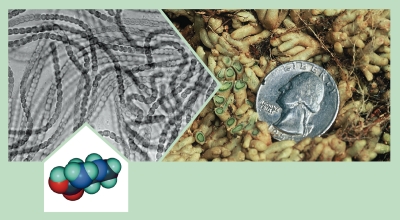
BMAA is produced by 95% of the genera of cyanobacteria tested, including Nostoc, which grows in the roots of the cycad tree and appears as the green lining in the cutaway roots pictured above. Molecular structure: © Scimat/Photo Researchers, Inc.; all other images: Paul A. Cox

Cox’s colleague Sandra Banack, then a biology professor at California State University, Fullerton, was working with Cox in Hawaii analyzing Mariana flying fox skins to determine whether they contained BMAA. “We decided if [BMAA] wasn’t in the bats, we’d just move on,” says Cox. In a way, Cox says, he hoped BMAA wouldn’t be the triggering compound, remembering the ridicule encountered by Spencer in the 1980s. But one night around 2:00 a.m., he recalls, “Sandra called me from the lab, and I picked up on the first ring. She said, ‘We got it.’”

About the same time, Cox and Banack made another advance, discovering that BMAA was produced by cyanobacteria that lived as symbionts in specialized roots of the cycads.^12^ They tested BMAA in various organisms along the food chain and found samples of Mariana flying fox skin had exorbitant BMAA levels averaging 3,556 µg/g.^13^ This was 10,000 times more than was found in free-living cyanobacteria and 3 times as much as in the fleshy cycad seed coat eaten by the bats, supporting the idea of biomagnification (in which a contaminant—usually a fat-soluble compound—accumulates in an organism).^12^ But the biggest surprise came when they tested human brains in a blinded study. They found high BMAA concentrations not just in the brains of all ALS-PDC patients tested but also in the brains of Canadians who died of Alzheimer disease (AD)—yet not in age-matched controls.^14^ If BMAA was produced by cyanobacteria on Guam, how could people in Canada be exposed?

## A Cyanobacterial Neurotoxin

Because cyanobacteria photosynthesize, scientists once classified them as algae—and many people still refer to them as blue-green algae—but modern genetics reveals a separate evolutionary lineage. Cyanobacteria either form symbiotic relationships with other organisms or live alone in fresh and marine waters, where they can erupt in sprawling and often toxic blooms associated with high nutrient inputs such as fertilizer runoff. They also are found in desert crusts, where they spring to life with seasonal rains. The incidence of cyanobacterial blooms has increased worldwide and may grow even more widespread with warming climates.^15^

In a 2005 article in *Proceedings of the National Academy of Sciences*, Cox and several colleagues reported testing 30 laboratory strains of cyanobacteria and finding that 95% of the genera tested produced BMAA.^16^ “We realized that once we published this result, it was going to shake a lot of trees,” says Cox. It could mean a paradigm shift for a field that has invested a lot of money studying the genetics of ALS rather than environmental triggers.

Only 5–10% of ALS, AD, and Parkinson disease (PD) cases are due to inherited genetic mutations, says Walter Bradley, an ALS expert and former chairman of neurology at the University of Miami Miller School of Medicine. “Hundreds of millions of dollars have been spent looking for predisposing genes, but . . . there is really a need to concentrate much more on environmental toxicants,” Bradley says.

“Big Pharma has spent big money to come up with new medications targeted against the best mechanisms that the scientific community has tested,” says Deborah Mash, a neurologist at the University of Miami Miller School of Medicine and director of the Miami Brain Bank. Collaborating with Bradley, she replicated Cox’s brain study, finding BMAA in the brains of AD, PD, and ALS victims but not in controls.^17^ She also showed BMAA crosses the blood–brain barrier in rats. In these studies, they found that the molecule takes longer to get into the brain than into other organs, but once there, it gets trapped in proteins, forming a reservoir for slow release over time.^18^^,^^19^

**Figure:**
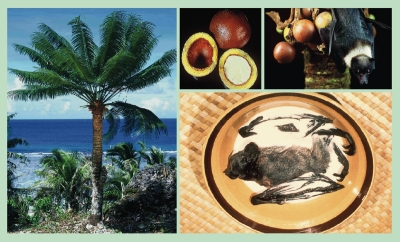
The seeds of the cycad are used as food and medicine by the indigenous Chamorro people of Guam. They are also eaten by bats and feral pigs that are consumed by the Chamorro. The resulting heavy dietary intake of BMAA has been linked with a constellation of neurodegenerative symptoms known locally as lytico-bodig. All images: Paul A. Cox

Once Cox realized that BMAA may be involved in multiple neurodegenerative diseases, he left Hawaii to establish the Institute for EthnoMedicine in Jackson Hole, Wyoming, where he was joined by Banack and later by James Metcalf, a cyanobacterial expert. While Banack got busy with lab work, Cox raised funds for the institute’s research and established a loose consortium of scientists around the world—epidemiologists, neurobiologists, and ecologists. They meet once a year to discuss research findings and directions for future research.

Cox has spent the bulk of his research efforts focusing on ALS, in part for humanitarian reasons; ALS strikes healthy, predominantly middle-aged people seemingly at random, and, of the major neurodegenerative diseases, it has the least hope for treatment and survival (in clinical trials, the only FDA-approved treatment^20^ for ALS offered approximately 3 extra months of life, although improved treatment protocols may extend this time).

ALS affects motor neurons, the longest cells in the body. Although mental capabilities stay intact, ALS paralyzes patients, often from the periphery inward, and most patients die within 3 years when they can no longer breathe or swallow. At any given time, an estimated 30,000 ALS cases exist in the United States^21^ (compared with 5.4 million AD patients^22^ and 500,000 PD patients^23^), but lifetime risk in this country is estimated at approximately 1 in 350 for men and 1 in 450 for women.^24^ Only 10% of cases are thought to be inherited (these are termed “familial ALS”), with 15–20% of these linked to mutation in the SOD1 (superoxide dismutase) gene.^25^ The cause of the remaining 90% (termed “sporadic ALS”) remains unexplained.^26^

## Mistakes in Translation

A foundational aspect of Cox’s hypothesis—and the part that has proven the most contentious^10^^,^^27^^,^^28^^,^^29^^,^^30^—is that BMAA not only occurs as a free, water-soluble molecule but also gets bound into proteins. Since hydrolysis is necessary to release protein-bound BMAA,^31^ Cox suspects that other studies underestimated or missed BMAA altogether.^32^^,^^33^ BMAA is a nonproteinogenic amino acid, meaning it is not one of the 20 amino acids that make up proteins in all eukaryotic organisms.

Accumulation of BMAA in the proteins of nerve cells, which need to last a lifetime, would provide a mechanism for how the toxin might biomagnify. “The problem with neurons is they do not divide, as a general rule, so over time they accumulate damaged proteins, and once they reach a critical level, it causes the cell to undergo apoptosis [cell death],” explains Rachael Dunlop, a researcher with the Heart Research Institute in Sydney, Australia.

**Figure:**
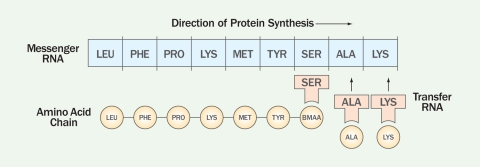
The genetic code in the messenger RNA holds the “recipe” for constructing a protein, and transfer RNA, which contains the matching code, attaches the correct amino acid. New research indicates BMAA can bind to serine (SER) transfer RNA and become part of the protein chain.^40^ Kenneth Rodgers, Matthew Ray/EHP

**Figure:**
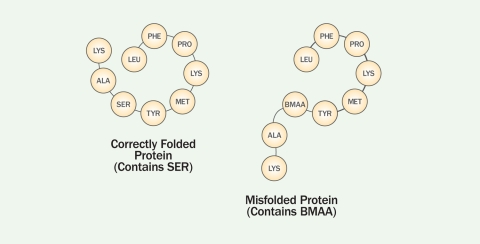
Once BMAA is incorporated into the chain, the protein can no longer fold properly. Clumps of misfolded proteins may form the aggregates that characterize neurodegenerative disease.^41^ Kenneth Rodgers, Matthew Ray/EHP

In rapidly dividing nonneuronal cells, Dunlop explains, damaged proteins that have formed aggregates are diluted into daughter cells. “These cells effectively get rid of their ‘junk’ by dilution,” she says. “Neuronal cells can’t do that and eventually get overwhelmed and die.” Accumulation could also explain how BMAA could plausibly lead to the telltale misfolded proteins observed in the brains of those who die of neurodegenerative disease.

But Cox and colleagues had no additional conclusive evidence linking BMAA and protein aggregates in the brain, and his idea came under fire. “The whole scientific world at that time thought that the machinery of cells would look at [BMAA] and say, ‘That’s not one of the twenty [proteinogenic] amino acids,’” says Bradley. Each amino acid has its own transfer RNA (tRNA) synthetase, a highly specific enzyme that picks up an amino acid and attaches it to the matching codon on the messenger RNA (mRNA) during translation, an early step in the process of protein synthesis. “There has been a gradual accumulation of research showing that not only does misincorporation of various nonproteinogenic amino acids occur, but it also can cause human and animal disease,” Bradley says.

In 2006 Susan Ackerman and colleagues published in *Nature* that misincorporation of the wrong proteinogenic amino acid, even at the background error rate (1 error per 1,000–10,000 codons), can lead to neurodegeneration in mice.^34^ Other research has revealed that organisms do, in fact, misincorporate nonproteinogenic amino acids.^4^^,^^35^ In 2002 Kenneth Rodgers, a senior lecturer at the University of Technology, Sydney, found that a range of nonproteinogenic amino acids can be incorporated into cell proteins by mammalian cells, among them the amino acid levodopa (l-DOPA), the treatment most commonly used for PD.^36^ Rodgers subsequently detected l-DOPA in brain proteins of treated PD patients.^37^^,^^38^ “We have sequenced proteins and shown that l-DOPA is incorporated into proteins in place of tyrosine,^39^” says Rodgers, who is now part of Cox’s consortium.

Evidence that BMAA isn’t just found in brain tissue but actually gets misincorporated into nerve cell proteins and that this causes protein misfolding and ultimately cell death was presented at the International ALS/Motor Neuron Disease (MND) Symposium in December 2011.^40^ Rodgers and Dunlop reported that the tRNA synthetase enzyme for the amino acid serine mistakenly picks up BMAA and incorporates it into proteins *in vitro*. Consequent autofluorescence indicated that the proteins misfolded, and the cells died.^41^

## Multiple Modes of Neurotoxicity

In most proteins, the hydrophilic (water-loving) parts stay on the outside while the hydrophobic (water-repelling) parts stay on the inside of the structure, but damage or mistakes in translation, such as misincorporation of BMAA, can cause the hydrophobic parts of the protein to end up exposed. These sticky parts adhere to other misformed proteins, forming “aggregates,” a telltale sign of neurodegenerative disease.^42^ The formation of small aggregates seeds the formation of larger, more toxic aggregates in a sort of chain reaction that prevents the cells from functioning effectively.^43^

Recent ALS research has focused on the role of TAR DNA-binding protein 43 (TDP-43) in neurodegeneration.^44^ “TDP-43 has been found in protein aggregates from patients affected by both familial and sporadic forms of ALS, so even without the mutated gene, an improperly functioning TDP-43 can contribute to disease,” says Dunlop. “If the misincorporation of BMAA in place of serine into TDP-43 causes the protein to misfold or not function correctly, this could contribute to ALS. We are not naïve enough to think this is the only catalyst for sporadic ALS—there are likely several processes that need to come together to lead to motor neuron dysfunction and eventually death—but it’s a clue.”

“The recent finding that BMAA is mistaken for serine by tRNA synthetase during protein formation opens up a plethora of potential new studies in lab models of ALS, from yeast to mice, to see whether the formation of protein aggregates—the classic ‘hallmarks’ of motor neuron degeneration—can be replicated,” says Brian Dickie, director of research at the Motor Neurone Disease Association. “We held a similar session at the 2008 ALS/MND Symposium,^45^ and I was struck by how many more research groups are now working in this field. There is definitely greater acceptance that this avenue should be pursued.”

In addition to protein misincorporation, high levels of unbound BMAA can continually overstimulate glutamate receptors on cells, leading to neuronal injury.^46^ Marquette University biology professor Doug Lobner found that BMAA and methylmercury—a common pollutant in seafood—both deplete glutathione, the main endogenous antioxidant in the body, and they act synergistically to harm nerve cells.^47^^,^^48^ Depletion of glutathione increases free radical damage that is known to occur in neurodegenerative diseases and has been linked to ALS in a transgenic SOD1 mouse model, says Lobner.^49^ Because BMAA and methylmercury can both turn up in waterways, this synergistic action could pose a grave problem.

The jury is still out on how multiple variables work together to cause neurodegeneration. “There are genetic variables. There are environmental variables. There are human health variables. This is probably a smorgasbord of bad things that occur to you in your life,” says Mash. “Maybe it’s oxidative stress. Maybe it’s BMAA getting misincorporated, and you’re building up a bunch of junky proteins in the cell. And maybe you’re putting stress on the mitochondria, and the mitochondria are making more reactive oxidative species, and now you’ve got a one–two punch on the cell.”

## Other Lines of Inquiry

While the mechanisms of BMAA toxicity are being worked out, ecological work has provided other lines of evidence. Mash collaborated with algal ecologist Larry Brand of the University of Miami Rosenstiel School of Marine and Atmospheric Science to test for BMAA in sea life from Florida coastal waters, including Florida Bay, which has a massive recurring cyanobacterial bloom.^50^ Species low on the food chain, including pink shrimp and blue crab—both of which are eaten by humans—had high BMAA levels, comparable to the bat skins from Guam (one crab had 6,976 µg/g).^51^ Mash’s laboratory has also found BMAA in several shark species in unpublished research. Brand wants to test farm-raised shrimp since, he says, they grow in ponds flush with cyanobacteria.

Preliminary data also revealed BMAA in dolphin brains.^52^ “We’re interested in dolphins because they eat the same kind of seafood that we do,” says Brand. “I was pretty skeptical we’d see anything. From a chemical point of view, you really would not expect BMAA to biomagnify in the food chain.” It turned out 5 of 6 dolphin brains sampled contained BMAA; however, the cause of death—impact by a boat—was known only for the sixth dolphin, which had no detectable BMAA in its brain.

An important area of future research involves potential exposure routes in addition to seafood consumption. Some agricultural fields are irrigated from water bodies covered in cyanobacterial blooms, raising the potential that BMAA could get into milk, meat, or vegetables. Dan Dietrich, a toxicology professor at the Universität Konstanz in Germany, reported finding unspecified large quantities of BMAA in commercially available blue-green algae dietary supplements, including *Spirulina* and *Aphanizomenon flos–aquae,*^53^ a finding that has not been replicated. Cox isolated BMAA from desert crusts collected throughout Qatar and suggested that not only might the cyanotoxin have contributed to higher rates of ALS in Gulf War veterans but also that inhalation of BMAA-containing dust could be a concern elsewhere.^54^

Drinking water could be a potential exposure route, as well. Lake Houston, which provides drinking water to Houston, Texas, tested positive for BMAA in the fall of 2011.^55^ A study of the effectiveness of water treatment techniques on BMAA removal showed that sand filtration, powdered activated carbon, and chlorination were effective at removing BMAA—at least at the labora tory scale—with flocculation less so.^56^ No study has tested the effectiveness of real-world water treatment methods at BMAA removal, and at the present time, no food or drinking water supplies are tested for BMAA, although Cox suggests that monitoring would be prudent.^16^ Researchers at the Institute for EthnoMedicine recently developed an antibody that detects BMAA, which they imagine being incorporated into a commercial test and a BMAA-removing water filter.

Epidemiology provides another important line of evidence supporting the BMAA hypothesis. Dartmouth-Hitchcock Medical Center neurologist Elijah Stommel and colleagues used geographic information system (GIS) software to map ALS cases and lakes with a history of cyanobacterial blooms in New Hampshire. They found that people living within a half-mile of cyanobacterially contaminated lakes had a 2.32-times greater risk of developing ALS than the rest of the population; people around New Hampshire’s Lake Mascoma had up to a 25 times greater risk of ALS than the expected incidence.^57^ Although BMAA was found in water samples from other lakes, the researchers did not detect it in Lake Mascoma samples, perhaps, they suggest, because of the small amount of cyanobacteria collected on sampling filters. Nevertheless, says Stommel, “Our GIS mapping is clearly showing clusters in proximity to [harmful algal blooms].” He and his team added more patients to their database and are preparing 2 papers for submission in the near future.

## Connecting Dots

Scientists around the world continue to research different aspects of the hypothesis. Scientists in Sweden found that newborn rats treated with BMAA showed early neurotoxicity and impaired learning and memory as adults.^58^ Others are investigating high BMAA levels in oysters off the coast of southern France, where an ALS cluster occurs.

In 2010 the National Toxicology Program (NTP) began studies in rat and mouse models to determine, among other things, whether BMAA accumulates in the brain and other tissues and rates of clearance from those tissues. If accumulation is demonstrated, mechanistic studies may be designed to further characterize the neurotoxic potential of BMAA. Preliminary results from the NTP work will be presented at the March 2012 annual meeting of the Society of Toxicology.

“I don’t know if the BMAA hypothesis is true,” says Mash. “I know we’ve measured BMAA in the brain, but proximity is not causality. You have to have full mechanistic underpinnings [to demonstrate causality], and that’s going to take a lot of money. It’s going to take epidemiologic studies. It’s going to take other cell culture models to really explain how this could work. And the big ticket is going to be environmental studies.”

Among the most promising development are 2 drugs on the horizon that may help ALS patients. Cox and colleagues hope to develop a drug to potentially stop BMAA from being misincorporated, and Adeona Pharmaceuticals has begun Phase II and III clinical trials for a zinc-based drug^59^ that has already showed promise at slowing ALS progression, albeit with a very small sample size.^60^

Since many more people may be exposed to BMAA than succumb to neurodegenerative diseases, Cox suspects that vulnerability may reflect a gene–environment interaction. If BMAA increases the misfolding of aggregate-prone proteins such as TDP-43, this could represent such a gene–environment interaction and explain how a single environmental factor such as BMAA could precipitate ALS-, PD-, and AD-like illness that observed on Guam.

However, no one has yet investigated a genetic basis to BMAA vulnerability. In the meantime, Cox and colleagues suggest that people should take the threat seriously. “We encourage water managers to take a closer look at cyanobacterial blooms,” he says. “We need to encourage places where there are commercial shellfish fisheries to pay attention to water quality.”

Much work remains to be done. Yet, says Bradley, “I don’t think there’s any question that the scientific basis of BMAA and its neurotoxicity is moving along at a very satisfying pace, and it is all concordant with the hypothesis.”
